# A comparative epidemiologic analysis of SARS in Hong Kong, Beijing and Taiwan

**DOI:** 10.1186/1471-2334-10-50

**Published:** 2010-03-06

**Authors:** Eric HY Lau, C Agnes Hsiung, Benjamin J Cowling, Chang-Hsun Chen, Lai-Ming Ho, Thomas Tsang, Chiu-Wen Chang, Christl A Donnelly, Gabriel M Leung

**Affiliations:** 1 School of Public Health, The University of Hong Kong, Pokfulam Road, Hong Kong; 2 Division of Biostatistics and Bioinformatics, Institute of Population Health Sciences, National Health Research Institutes, 35, Keyan Road, Zhunan, Miaoli County 35053, Taiwan; 3 Second Division of Centers for Disease Control, No 6, Linshen South Road., Taipei, Taiwan; 4 Centre for Health Protection, Department of Health, Government of the Hong Kong Special Administrative Region, 147C Argyle Street, Kowloon, Hong Kong; 5 MRC Centre for Outbreak Analysis and Modelling, Department of Infectious Disease Epidemiology, Imperial College, St Mary's Campus, Norfolk Place, London W2 1PG, UK

## Abstract

**Background:**

The 2002-2003 Severe Acute Respiratory Syndrome (SARS) outbreak infected 8,422 individuals leading to 916 deaths around the world. However, there have been few epidemiological studies of SARS comparing epidemiologic features across regions. The aim of this study is to identify similarities and differences in SARS epidemiology in three populations with similar host and viral genotype.

**Methods:**

We present a comparative epidemiologic analysis of SARS, based on an integrated dataset with 3,336 SARS patients from Hong Kong, Beijing and Taiwan, epidemiological and clinical characteristics such as incubation, onset-to-admission, onset-to-discharge and onset-to-death periods, case fatality ratios (CFRs) and presenting symptoms are described and compared between regions. We further explored the influence of demographic and clinical variables on the apparently large differences in CFRs between the three regions.

**Results:**

All three regions showed similar incubation periods and progressive shortening of the onset-to-admission interval through the epidemic. Adjusted for sex, health care worker status and nosocomial setting, older age was associated with a higher fatality, with adjusted odds ratio (AOR): 2.10 (95% confidence interval: 1.45, 3.04) for those aged 51-60; AOR: 4.57 (95% confidence interval: 3.32, 7.30) for those aged above 60 compared to those aged 41-50 years. Presence of pre-existing comorbid conditions was also associated with greater mortality (AOR: 1.74; 95% confidence interval: 1.36, 2.21).

**Conclusion:**

The large discrepancy in crude fatality ratios across the three regions can only be partly explained by epidemiological and clinical heterogeneities. Our findings underline the importance of a common data collection platform, especially in an emerging epidemic, in order to identify and explain consistencies and differences in the eventual clinical and public health outcomes of infectious disease outbreaks, which is becoming increasingly important in our highly interconnected world.

## Background

The 2002-2003 SARS outbreak infected 8,422 individuals leading to 916 deaths in eight affected areas [1]. The first case was identified on 16 November 2002 in the southern Chinese city of Foshan [2]. The epidemic then spread within Guangdong province before a large superspreading event in Hong Kong seeded the global outbreak [3]. On 5 July 2003, the World Health Organization (WHO) announced the last affected area Taiwan to be transmission free and declared the last human-to-human transmission chain successfully interrupted [4]. Hong Kong, mainland China and Taiwan carried the largest disease burden as well as marked the most important milestones of the global outbreak. However, due to geo-political factors beyond the remit of public health, a consolidated account of their collective experience has not previously been documented.

Except for a few sporadic case reports mostly involving laboratory mishaps [5-7], there has not been sustained human-to-human transmission since 2003. However, the re-emergence of SARS remains a distinct possibility given that similar viruses continue to be isolated in potential animal reservoirs including bats [8,9] and palm civets [10], and trading of the latter continues in Guangdong province despite its official ban [11]. Moreover, reemergence in humans could result from an unknown animal reservoir, or via an intermediary animal host. In addition, among the three affected regions in Greater China, the disease had apparently different epidemiological characteristics whereas the populations share the same gene pool and the same viral clade [12]. These apparent epidemiologic heterogeneities despite similar host and viral genotypes as well as the preparedness imperative for a possible return of SARS motivated the present comparative analysis of the three regions, based on an integrated database of 3336 probable cases, which is the largest single repository of SARS patients constructed to date. We present comprehensive estimates of epidemiologic parameters of interest, and we also investigate potential explanations for the observed discrepancies between regions.

## Methods

### Sources of data

We analyzed a combined database of SARS patients from Hong Kong (n = 1755), Beijing (n = 917) and Taiwan (n = 664). Clinical, demographic and epidemiologic details for SARS patients from each region were collected, coded and anonymized for analysis. All analyses were based on patients who satisfied the WHO definition for 'probable cases' [13]. In Beijing, clinical presentation, exposure history, blood test, chest CT and X-ray scan were also reviewed in additional to the WHO definition.

Our database on all 1755 probable cases in Hong Kong was derived from the Department of Health master list and the Hospital Authority eSARS system as previously described [14].

Data on 917 of the 2521 probable cases in Beijing [15], who were either directly admitted or transferred to Xiao Tang Shan Hospital (XTS Hospital, n = 680), the No. 302 People's Liberation Army Hospital (Hospital 302, n = 111) or the No. 309 People's Liberation Army Hospital (Hospital 309, n = 126) were included. Clinical information was reviewed by a panel of experts in the earlier stage of the epidemics and furthermore a standardized report form was used after May as previously described [15]. Data were extracted by detailed chart review in each hospital following a standard protocol. Hospital 309 admitted many of the earliest SARS patients and reached full capacity by early April. SARS patients were then sent to Hospital 302, a hospital specializing in infectious diseases. The severity and infectiousness of SARS prompted the Beijing government to build the XTS Hospital in eight days, opening on 1 May 2003. Data on comorbidities, and symptoms were unavailable for patients in Hospital 309.

Similarly, we captured the corresponding data from all 664 probable cases in Taiwan who provided the information through standardized screening questionnaires at emergency rooms and on admission. Data were collected from all hospitals and integrated into a dataset officially maintained by the Centers for Disease Control, Taiwan.

### Statistical Analysis

We compared the characteristics of SARS patients by region in terms of demographic and clinical variables such as age, sex and health care worker (HCW) status, presence of pre-existing comorbid conditions (including ischemic heart disease, cerebrovascular disease, cancer, diabetes, chronic renal failure, chronic liver disease and asthma) and whether the patients were admitted before symptom onset. We estimated the case fatality ratios (CFRs) within subgroups of each of these characteristics. We calculated the age- and sex-standardized CFRs and associated exact binomial 95% confidence intervals based on the World Standard Population [16]. We fitted multivariable logistic regression models, controlled for variables such as sex, age, health care worker status, preexisting comorbid conditions and nosocomial infection, for each region on all data allowing us to estimate the adjusted odds ratios of mortality between regions. In the regression models we excluded all 126 patients from Hospital 309 for whom data on pre-existing comorbid conditions were not available and also other two, one and five patients from Hong Kong, Beijing and Taiwan respectively with unknown age, pre-existing comorbid conditions, onset date or admission date. To avoid extreme values in the adjusted odds ratio for the age effect, we chose the middle age group as the reference group. For Hong Kong patients, we also tested the effect of residence in Amoy Garden where a severe outbreak occurred in which many patients presented with diarrhea [17].

Incubation period was estimated on patients with dates of exposure to a potentially infected individual and a symptom onset date, using both non-parametric and parametric methods allowing for interval censoring [18]. We plotted the epidemic curves in each region, and examined the changes in onset-to-admission distribution over the course of the epidemic.

We identified factors which might have affected the onset-to-death and onset-to-discharge distribution and compared the differences across the regions. The onset-to-death and onset-to-discharge periods were firstly log-transformed and then fitted by linear regression separately to the same variables as in the previous model.

Finally, we examined regional differences in symptoms at presentation. All analyses were conducted in R version 2.3.1 [19].

## Results

Additional file 1 shows patient characteristics by region and the associated crude CFRs. Patients in Hong Kong were on average older (mean = 43.5 years) than those in Beijing (34.7 years) but younger than those in Taiwan (47.2 years). Case fatality was consistently and strongly age-dependent. Approximately one-third of Taiwan SARS patients had pre-existing comorbid conditions, which was much greater than Hong Kong (20%) and Beijing (4%, based on available data), and in each region the CFRs were markedly elevated for those patients with comorbidities.

Overall, the crude CFRs differed substantially across regions (Additional file 1). After standardizing for age and sex, the case fatality ratios converged somewhat, suggesting that some of the crude differences were due to the different patient demographic profiles particularly in patients' ages between the three regions.

Additional file 1 also displays the adjusted odds ratios of death for each predictor based on data from each region. The adjusted results confirm that older age, non-HCWs, presence of pre-existing comorbid conditions and admission before symptom onset were strongly associated with mortality. In the combined model (Additional file 2), the adjusted odds ratios (95% confidence intervals) of case fatality for Beijing and Taiwan were 0.18 (0.11 to 0.29) and 1.64 (1.29 to 2.07) compared to Hong Kong. When the XTS Hospital patients were excluded, the adjusted odds ratios (95% confidence intervals) of case fatality for Beijing was 0.98 (0.46 to 2.09) compared to Hong Kong. We also fitted a logistic regression analysis to test for any differential effects of sex, age, health care worker status, pre-existing comorbid conditions and nosocomial acquisition between Hospital 302 and XTS Hospital. All the first-order interaction effects were found to be insignificant. All main and interaction effects with residence in Amoy Garden were also found to be insignificant.

**Figure 1:**
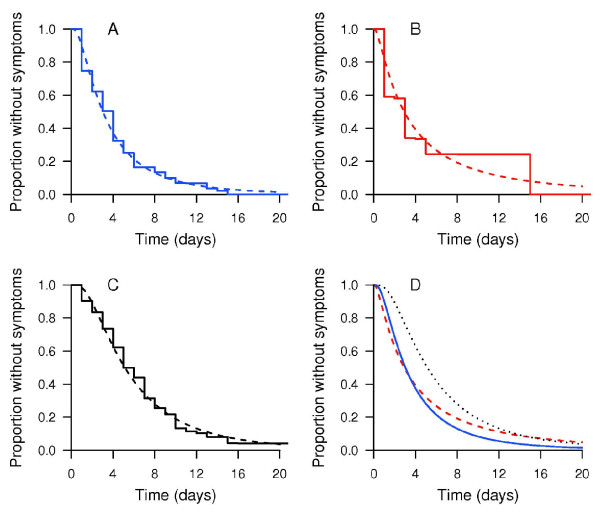
**Estimated incubation distribution**. (A) Hong Kong (n = 168), (B) Beijing* (n = 97) and (C) Taiwan (n = 156) using a non-parametric method (solid line) and fitted to a lognormal distribution (dashed line). (D) Comparison of estimated incubation distributions fitted to a lognormal distribution in Hong Kong (solid line), Beijing (dashed line), and Taiwan (dotted line). *The large steps seen in the non-parametric estimate in Beijing are aretefacts due to the small sample size.

Data on exposure and onset times were available for 168 patients in Hong Kong, 97 patients in Beijing and 210 patients in Taiwan. Fitted lognormal distributions showed good fit compared to non-parametric estimates of the incubation distribution (Figure 1). Based on the lognormal distributions, the estimated means (standard deviations) of the incubation distributions were 4.4 days (4.6 days) in Hong Kong, 5.7 days (9.7 days) in Beijing and 6.9 (6.1 days) in Taiwan. The 25, 50, 75 and 95^th ^percentiles were 2, 3, 5 and 12 days in Hong Kong, 1, 3, 6 and 20 days in Beijing and 3, 5, 9 and 18 days in Taiwan.

**Figure 2:**
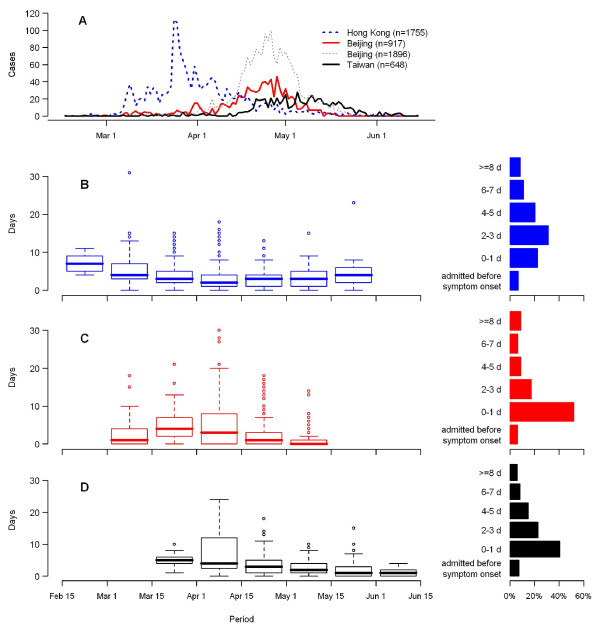
**Epidemiological characteristics in Hong Kong, Beijing and Taiwan**. (A) Epidemic curve for Hong Kong, Beijing (based on our data and those from a Beijing dataset [15]), and Taiwan. B-D*: Time from onset to admission distribution for each 2-week period and onset-to-admission distribution for (B) Hong Kong, (C) Beijing and (D) Taiwan. * Patients admitted before symptom onset and periods with fewer than 10 cases were excluded.

Figure 2A shows the epidemic curve in each region, supplemented with the overall epidemic curve in Beijing [15]. The epidemic in Hong Kong occurred mainly in late March and early April 2003, preceding the outbreaks in Beijing and Taiwan by approximately one month. Figure 2B, C and 2D show box plots of the time from onset to admission during different calendar time periods and the overall distributions of onset-to-admission intervals. Within each region, the onset-to-admission times decreased throughout the main part of all three epidemics, while onset-to-admission times appeared to increase towards the end of the Hong Kong epidemic. Excluding the patients admitted before symptom onset, the mean delay from onset to admission were 3.6, 2.7 and 2.8 days respectively for Hong Kong, Beijing and Taiwan. A relatively large proportion of patients were admitted to hospital within one day after symptom onset in both Beijing and Taiwan, and Taiwan had a higher proportion of patients admitted before symptom onset. In Hong Kong and Taiwan, patients admitted before symptom onset had a much higher CFR (53% and 70% respectively, see Additional files 3 and 4). Hong Kong and Taiwan showed a decreasing gradient with prolonged delay from first symptoms to hospitalization until about one week whereas the opposite appeared to be the case in Beijing. Within Beijing, there was a clear difference in both onset-to-admission distributions and CFRs among hospitals although the trends were mostly consistent (see Additional file 4).

The median onset-to-death periods in Hong Kong, Beijing and Taiwan were 21, 24 and 10 days respectively, while the median onset-to-discharge periods were 23, 36 and 19 days respectively. We also examined factors affecting the length of these periods (see Additional file 5). Patients in the youngest age group and those who had acquired SARS nosocomially had significantly shorter onset-to-death periods, while non-health care workers and those with pre-existing comorbid conditions had marginally shorter onset-to-death periods. When comparing across the three regions, Hong Kong and Beijing patients had similar onset-to-death periods, but those of patients in Taiwan were significantly shorter. As a sensitivity analysis, we show the factors affecting onset-to-death and onset-to-discharge excluding data from XTS Hospital (see Additional file 6).

Regarding the onset-to-discharge period, younger patients were found to have shorter lengths of stay in hospital on average. We found statistically significant interaction effects between HCW status and location and between pre-existing comorbid conditions and location, where HCWs in Taiwan had longer onset-to-discharge periods (acceleration factor = 1.35, 95% CI: 1.17, 1.55), while those with pre-existing comorbid conditions in Taiwan had shorter periods (acceleration factor = 0.87, 95% CI: 0.75, 1.02). After adjustment for the above factors, Beijing had a significantly longer onset-to-discharge period comparing with Hong Kong, while Taiwan had a significantly shorter period.

**Figure 3:**
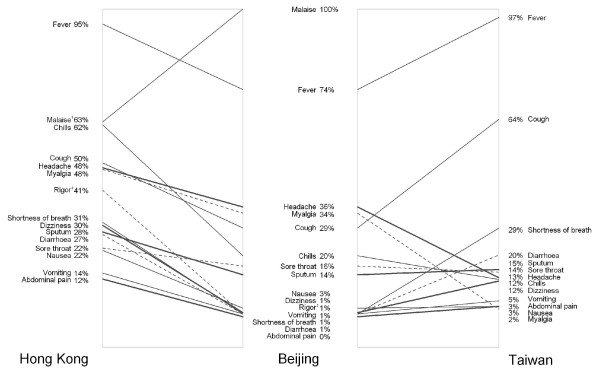
**Proportion of cases showing different symptoms on presentation in Hong Kong, Beijing (Hospital 302 only^†^) and Taiwan**. * Labels on the vertical axis have been jittered for better presentation. ^† ^Detailed symptom data were missing for Hospital 309 patients. ^‡ ^Malaise and rigor were not documented in Taiwan.

Figure 3 summarizes symptoms at the time of presentation in Hong Kong, Beijing (Hospital 302 only) and Taiwan. While the prevalence of the different symptoms were similar in Beijing and Taiwan, the occurrence of most symptoms was typically higher in Hong Kong. Fever was the most common presenting symptom in all three regions. Dizziness, rigor, shortness of breath as well as gastro-intestinal symptoms including diarrhea, vomiting and abdominal pain were rarely reported in Beijing, on the other hand every patient in Beijing reported malaise.

## Discussion and Conclusions

This set of analyses is the most comprehensive to date, based on data accounting for 40% of the global case load. Simultaneously analysing data from various affected areas in Greater China together revealed consistencies and explained heterogeneities that would have otherwise remained unexplored. Our combined database is unique in that of the three regions, only mainland China is a member of the WHO whereas Hong Kong is an observer and Taiwan is a non-member. Such geo-political influences have posed an almost insurmountable challenge to official supranational agencies in pulling together the respective databases [20]. Database construction during an outbreak is critical to underpin analyses that will inform policy decisions regarding both disease control and treatment of infected patients. Key to the utility of such databases is their scope, format, accuracy and timeliness. While we have largely succeeded on the first two counts, the fact that we are only reporting results seven years after SARS receded into its animal reservoir points to an urgent deficiency in the global public health research infrastructure. Nevertheless, this report underscores the positive steps forward in recent years.

Our findings confirm the widely reported heterogeneous CFRs in the three regions [1,21,22]. However, discounting the highly selected XTS Hospital sample, the Beijing CFR approximated that of Hong Kong. On the other hand, Taiwan had a 64% higher adjusted case fatality compared to Hong Kong. Clearly one of the most important explanations for the observed low case fatality ratio in our subset of patients from Beijing is the inherent selection bias. While we studied 36% of the reported probable SARS cases in Beijing, from three of the largest SARS hospitals, our subset is by no means representative. The majority (74%) of our patients were from the XTS Hospital, a facility which was built especially to house SARS patients and opened halfway through the epidemic. Only one of the 680 inpatients was directly admitted, the remainder were transferred from other hospitals in Beijing. Although some of the XTS patients experienced symptom onset as early as the end of March, admission to XTS would have required survival until at least May 1, when XTS opened. Further, it is less likely that patients in serious condition (for example requiring mechanical ventilation) would have been transferred. As further confirmation of the systematic selection of the XTS sample, we found that patients admitted to XTS Hospital were epidemiologically biased, as shown by having shorter onset-to-admission periods and longer onset-to-discharge periods (data not shown).

An interesting related finding is that the relatively higher CFR also applied consistently to each subgroup including low risk groups such as younger patients and HCWs, which suggests that the presence of some common factors not included in our models (e.g. treatment protocols, hospital setting) may have independently increased CFRs in Taiwan. However, the treatment of choice in Hong Kong and Taiwan was the combined use of ribavirin and corticosteroids [14,23], and a large meta-analysis has shown that these treatments were likely ineffective [24]. We also observed similar patterns of CFRs for patients with the four most common comorbidities (diabetes, cardiovascular disease, ischaemic heart disease and chronic obstructive airways disease; data not shown) between Hong Kong and Taiwan and we did not observe exceptionally high case-fatality ratios among the patients with comorbidities in Taiwan. Hence the difference is probably not attributable to anti-viral or immunologic regimens but could have been related to different levels of general supportive care. Overall, our analysis found that age and pre-existing comorbid conditions were two major determinants of fatality, which is consistent with existing studies [14,25-30]. Male sex was significantly associated with increased risk of fatality in Hong Kong after adjustment for other important confounding factors, consistent with a previous study in Hong Kong that identified a sex effect in unadjusted analyses of aggregate data [31]. The reasons for an increase in risk of death among males remain unclear. In addition we found that admission before symptom onset, most likely due to nosocomial acquisition, was also significantly associated with higher fatality rate after adjustment for comorbidities in Taiwan [32].

In terms of epidemiologic parameters, the incubation period had mean 4.4, 5.7 and 6.9 days and a 95^th ^percentile of 12.4, 19.7 and 17.9 days in Hong Kong, Beijing and Taiwan respectively, with some degree of variation between regions [18]. Together they can reliably inform the diagnostician the likelihood of SARS in a suspected case and advise the public health practitioner the appropriate period of quarantine. While most countries used a ten-day quarantine period, based on early estimates of the incubation period [33], our findings suggest that a longer period may be appropriate if SARS were to reemerge [29]. However differences between regions in the incubation distribution, as well as in the onset to discharge/death distributions discussed below, may be due to different interpretations of disease onset between the regions.

As would be expected, our results show that onset-to-admission periods shortened throughout the epidemic (Figure 2) as public awareness improved and public health interventions were implemented [14,34,35]. However, in the three regions, there was no evidence that shorter onset-to-admission period resulted in lower fatality (see Additional file 3), possibly due to the lack of effective treatment and that viremia only peaks after seven to ten days [26].

Taiwan's shorter onset-to-death distributions, indicating quicker deterioration, coupled with the consistently higher fatality ratios for most subgroups raises the possibility that the management of these patients did not compare favorably with those in the other two regions, especially if we assume the viral agent and host genetic susceptibility were similar throughout. However without more detailed clinical data, for example levels of viral load or as a proxy lactate dehydrogenase at admission reflecting initial illness severity [14,29], it is not possible to discern here whether management and treatment, or some other factors, were responsible for the higher fatality ratios in Taiwan. Alternatively, differences in environment may be responsible for some observed differences, given the known associations between for example smoking and respiratory disease [36,37]. The relatively longer onset-to-discharge periods in Beijing could likely be explained by different clinical or official protocols [38].

Our description of the variability between regions in symptoms at presentation (Figure 3) is the first such comparison in the literature. The higher rates in Hong Kong of almost every symptom could be due to differences in reporting (or asking) behavior. This again points to the need for a universal information supply chain, from case and symptom definitions to guidelines in history taking and data coding, for newly emerging or resurging diseases of supranational interest. As previously noted all three areas analyzed here shared the identical viral strain, as well as the same ethnic gene pool, so it is less likely that differences are due to the infective agent or host genetic factors. Although there are differences in the absolute rates of the various symptoms, we note that a previously derived clinical prediction rule for SARS [39] has been validated on patients from Taiwan [40].

Finally, a few limitations bear mention. As in most previous studies [14,29,41,42], our analysis was based on probable cases of SARS according to the WHO definition [13] rather than laboratory confirmed cases because the latter definition may be biased toward including more survivors, particularly among the earlier cases [14]. Furthermore, rapid diagnostic SARS tests were not available until fairly late in the epidemic and had poor sensitivity for detecting the disease [43,44]. The WHO case definitions may have been applied differently in the three regions, or a different percentage of probable cases might have been caused by other pathogens, which may have led to the observed differences. Furthermore, while SARS patients in Beijing were classified according to the WHO definition, but a more recent detailed case review has found that some reported 'probable' SARS cases may have been misclassified [45]. We note that odds ratios should be interpreted with caution since they may be a poor approximation to relative risks with outcomes that have a high prevalence. Asymptomatic and subclinical infections of SARS were not considered in our analysis, although there is strong evidence that very few existed [46]. A final limitation is that, as previously discussed, the Beijing patients in our database mostly were hospitalized in XTS Hospital and were found to be epidemiologically different from patients in other hospitals, which makes it difficult to generalize our results to all patients in Beijing.

## Competing interests

The authors declare that they have no competing interests.

## Authors' contributions

BJC and GML conceived and designed the study. EHYL, CAH and BJC performed the statistical analysis. EHYL, CAH, BJC and GML analyzed and interpreted the data. EHYL and BJC drafted the manuscript. CHC, TT and CWC provided the study materials or patient data. CAH, CAD and GML obtained the funding. CHC, LMH and CWC collected and collated the data. All authors critically reviewed the manuscript, read and approved the final manuscript.

## Pre-publication history

The pre-publication history for this paper can be accessed here:

http://www.biomedcentral.com/1471-2334/10/50/prepub

## Supplementary Material

Additional file 1 **Characteristics of SARS patients in Hong Kong, Beijing and Taiwan**. The associated case-fatality ratios and adjusted odds ratios (95% confidence intervals) are also reported. CFR, case fatality ratio; AOR, adjusted odds ratio; CI, confidence interval. * Patients with unknown age, pre-existing comorbid conditions or admission date were excluded. ^† ^Adjusted for sex, age, health care worker status, preexisting comorbid conditions and nosocomial infection. ^‡ ^Data on final outcome were not available for 12 patients in Taiwan and were excluded for analysis. ^§ ^The estimates were not shown as there was not more than 2 deaths in these age groups out of a relatively large number of patients. ^|| ^Based on the WHO World Standard Population distribution [16]. Click here for file

Additional file 2 **Characteristics of SARS patients in Hong Kong, Beijing and Taiwan (pooled data)**. The associated case-fatality ratios and adjusted odds ratios (95% confidence intervals) are also reported. CFR, case fatality ratio; AOR, adjusted odds ratio; CI, confidence interval. * Data on final outcome were not available for 12 patients in Taiwan and were excluded for analysis. Patients with unknown age, pre-existing comorbid conditions or admission date were excluded from multivariable logistic regression models. ^† ^Adjusted for sex, age, health care worker status, preexisting comorbid conditions, nosocomial infection and region. ^‡ ^Based on the WHO World Standard Population distribution [16]. Click here for file

Additional file 3 **Case fatality ratio by different onset-to-admission periods, Hong Kong, Beijing and Taiwan**. CFR, case fatality ratio; CI, confidence interval. * Excluding 16 patients with unknown admission dates or discharge outcome. Click here for file

Additional file 4 **Case fatality ratio by different onset-to-admission periods in Beijing, XTS Hospital, Hospital 302 and Hospital 309**. CFR, case fatality ratio; CI, confidence interval. Click here for file

Additional file 5 **Factors affecting the onset-to-death and onset-to-discharge period of SARS patients in Hong Kong, Beijing and Taiwan**. CI, confidence interval. * The acceleration factor is computed as exp(β). It indicates the relative increase (>1) or decrease (<1) in the median time from onset of symptoms to death or discharge. ^† ^also adjusted for interaction between location with admission before symptom onset. ^‡ ^also adjusted for interaction between location with health care worker and pre-existing comorbid conditions. Click here for file

Additional file 6 **Factors affecting the onset-to-death and onset-to-discharge period of SARS patients in Hong Kong, Beijing (restricted to Hospitals 302 and 309 only and Taiwan**. CI, confidence interval. * The acceleration factor is computed as exp(β). It indicates the relative increase (>1) or decrease (<1) in the median time from onset of symptoms to death or discharge. ^† ^also adjusted for interaction between location with admission before symptom onset. ^‡ ^also adjusted for interaction between location with health care worker and pre-existing comorbid conditions. Click here for file

## References

[B1] Summary of probable SARS cases with onset of illness from 1 November 2002 to 7 August 2003 http://www.who.int/csr/sars/country/country2003_08_15.pdf

[B2] Zhong NS Zheng BJ Li YM Poon XZH Chan KH Li PH Tan SY Chang Q Xie JP Liu XQ Epidemiology and cause of severe acute respiratory syndrome (SARS) in Guangdong, People's Republic of China, in February, 2003 Lancet 2003 362 1353 1358 10.1016/S0140-6736(03)14630-2 14585636 PMC7112415

[B3] CDC SARS Investigative Team Update: outbreak of severe acute respiratory syndrome--worldwide, 2003 MMWR Morb Mortal Wkly Rep 2003 52 269 272 12729074

[B4] SARS outbreak contained worldwide http://www.who.int/mediacentre/news/releases/2003/pr56/en/

[B5] Escudero IH Chen MI Leo YS Surveillance of severe acute respiratory syndrome (SARS) in the post-outbreak period Singapore Med J 2005 46 165 171 15800722

[B6] Watts J SARS under control, but lab-safety questions remain Lancet 2004 363 1780 10.1016/S0140-6736(04)16344-7 15174476 PMC7135262

[B7] Heymann DL Aylward RB Wolff C Dangerous pathogens in the laboratory: from smallpox to today's SARS setbacks and tomorrow's polio-free world Lancet 2004 363 1566 1568 10.1016/S0140-6736(04)16234-X 15145625 PMC7135754

[B8] Chu DK Poon LL Chan KH Chen H Guan Y Yuen KY Peiris JS Coronaviruses in bent-winged bats (Miniopterus spp.) J Gen Virol 2006 87 2461 2466 10.1099/vir.0.82203-0 16894183

[B9] Li W Shi Z Yu M Ren W Smith C Epstein JH Wang H Crameri G Hu Z Zhang H Bats are natural reservoirs of SARS-like coronaviruses Science 2005 310 676 679 10.1126/science.1118391 16195424

[B10] Wang M Yan M Xu H Liang W Kan B Zheng B Chen H Zheng H Xu Y Zhang E SARS-CoV infection in a restaurant from palm civet Emerg Infect Dis 2005 11 1860 1865 16485471 10.3201/eid1112.041293 PMC3367621

[B11] Civet cats found at restaurants again http://www.chinadaily.com.cn/china/2007-02/14/content_808890.htm

[B12] Liu W Tang F Fontanet A Zhan L Wang TB Zhang PH Luan YH Cao CY Zhao QM Wu XM Molecular epidemiology of SARS-associated coronavirus, Beijing Emerg Infect Dis 2005 11 1420 1424 16229772 10.3201/eid1109.040773 PMC3310602

[B13] Case Definitions for Surveillance of Severe Acute Respiratory Syndrome (SARS) http://www.who.int/csr/sars/casedefinition/en/

[B14] Leung GM Hedley AJ Ho LM Chau P Wong IO Thach TQ Ghani AC Donnelly CA Fraser C Riley S The epidemiology of severe acute respiratory syndrome in the 2003 Hong Kong epidemic: an analysis of all 1755 patients Ann Intern Med 2004 141 662 673 15520422 10.7326/0003-4819-141-9-200411020-00006

[B15] Liang W Zhu Z Guo J Liu Z Zhou W Chin DP Schuchat A Beijing Joint SEG Severe acute respiratory syndrome, Beijing, 2003 Emerg Infect Dis 2004 10 25 31 15078593 10.3201/eid1001.030553 PMC3092360

[B16] Ahmad OB Boschi-Pinto C Lopez AD Murray CJ Lozano R Inoue M Age Standardization of Rates: A New WHO Standard 2000 Geneva: World Health Organization

[B17] Lee SH The SARS epidemic in Hong Kong J Epidemiol Community Health 2003 57 652 654 10.1136/jech.57.9.652 12933765 PMC1732583

[B18] Cowling BJ Muller MP Wong IO Ho LM Louie M McGeer A Leung GM Alternative methods of estimating an incubation distribution: examples from severe acute respiratory syndrome Epidemiology 2007 18 253 259 10.1097/01.ede.0000254660.07942.fb 17235210

[B19] R Development Core Team R: A Language and Environment for Statistical Computing 2008 Vienna: R Foundation for Statistical Computing

[B20] Editorial Public health versus political frontiers Lancet 2007 369 616 10.1016/S0140-6736(07)60286-4 PMC7135520 17321291

[B21] Chan KS Zheng JP Mok YW Li YM Liu YN Chu CM Ip MS SARS: prognosis, outcome and sequelae Respirology 2003 8 Suppl S36 40 10.1046/j.1440-1843.2003.00522.x 15018132 PMC7169213

[B22] Galvani AP Lei X Jewell NP Severe acute respiratory syndrome: temporal stability and geographic variation in case-fatality rates and doubling times Emerg Infect Dis 2003 9 991 994 12967499 10.3201/eid0908.030334 PMC3020622

[B23] Wang JT Sheng WH Fang CT Chen YC Wang JL Yu CJ Chang SC Yang PC Clinical manifestations, laboratory findings, and treatment outcomes of SARS patients Emerg Infect Dis 2004 10 818 824 15200814 10.3201/eid1005.030640 PMC3323212

[B24] Stockman LJ Bellamy R Garner P SARS: systematic review of treatment effects PLoS Med 2006 3 e343 10.1371/journal.pmed.0030343 16968120 PMC1564166

[B25] Ghani AC Donnelly CA Cox DR Griffin JT Fraser C Lam TH Ho LM Chan WS Anderson RM Hedley AJ Leung GM Methods for estimating the case fatality ratio for a novel, emerging infectious disease Am J Epidemiol 2005 162 479 486 10.1093/aje/kwi230 16076827 PMC7109816

[B26] Peiris JS Chu CM Cheng VC Chan KS Hung IF Poon LL Law KI Tang BS Hon TY Chan CS Clinical progression and viral load in a community outbreak of coronavirus-associated SARS pneumonia: a prospective study Lancet 2003 361 1767 1772 10.1016/S0140-6736(03)13412-5 12781535 PMC7112410

[B27] Chen KT Twu SJ Chang HL Wu YC Chen CT Lin TH Olsen SJ Dowell SF Su IJ Taiwan SRT SARS in Taiwan: an overview and lessons learned Int J Infect Dis 2005 9 77 85 10.1016/j.ijid.2004.04.015 15708322 PMC7110635

[B28] Donnelly CA Ghani AC Leung GM Hedley AJ Fraser C Riley S Abu-Raddad LJ Ho L-M Thach T-Q Chau P Epidemiological determinants of spread of causal agent of severe acute respiratory syndrome in Hong Kong Lancet 2003 361 1761 1766 10.1016/S0140-6736(03)13410-1 12781533 PMC7112380

[B29] Cowling BJ Muller MP Wong IOL Ho L-M Lo S-V Tsang T Lam TH Louie M Leung GM Clinical prognostic rules for severe acute respiratory syndrome in low- and high-resource settings Arch Intern Med 2006 166 1505 1511 10.1001/archinte.166.14.1505 16864761

[B30] Fowler RA Lapinsky SE Hallett D Detsky AS Sibbald WJ Slutsky AS Stewart TE Critically ill patients with severe acute respiratory syndrome JAMA 2003 290 367 373 10.1001/jama.290.3.367 12865378

[B31] Karlberg J Chong DS Lai WY Do men have a higher case fatality rate of severe acute respiratory syndrome than women do? Am J Epidemiol 2004 159 229 231 10.1093/aje/kwh056 14742282 PMC7110237

[B32] Chang IS Hsiung CA Wen CC Wu YJ Yang CC Non-parametric maximum-likelihood estimation in a semiparametric mixture model for competing-risks data Scand J Statist 2007 34 870 895

[B33] Severe acute respiratory syndrome--Singapore, 2003 MMWR Morb Mortal Wkly Rep 2003 52 405 411 12807088

[B34] Liang WN Liu M Chen Q Liu ZJ He X Pan Y Xie XQ Assessment of impacts of public health interventions on the SARS epidemic in Beijing in terms of the intervals between its symptom onset, hospital admission, and notification Biomed Environ Sci 2005 18 153 158 16131016

[B35] Cowling BJ Ho LM Leung GM Effectiveness of control measures during the SARS epidemic in Beijing: a comparison of the Rt curve and the epidemic curve Epidemiol Infect 2008 136 562 566 10.1017/S0950268807008722 17568476 PMC2870828

[B36] Brauer M Hoek G Van Vliet P Meliefste K Fischer PH Wijga A Koopman LP Neijens HJ Gerritsen J Kerkhof M Air pollution from traffic and the development of respiratory infections and asthmatic and allergic symptoms in children Am J Respir Crit Care Med 2002 166 1092 1098 10.1164/rccm.200108-007OC 12379553

[B37] Ponce de Leon A Anderson HR Bland JM Strachan DP Bower J Effects of air pollution on daily hospital admissions for respiratory disease in London between 1987-88 and 1991-92 J Epidemiol Community Health 1996 50 Suppl 1 s63 70 10.1136/jech.50.Suppl_1.s63 8758227 PMC1060891

[B38] Battling the spread of SARS, Asian nations escalate travel restrictions http://www.iht.com/articles/2003/04/12/a7_20.php

[B39] Leung GM Rainer TH Lau FL Wong IO Tong A Wong TW Kong JH Hedley AJ Lam TH A clinical prediction rule for diagnosing severe acute respiratory syndrome in the emergency department Ann Intern Med 2004 141 333 342 15326019 10.7326/0003-4819-141-5-200409070-00106

[B40] Ma MH Chen SY Chiang WC Su CP Chen WJ A clinical prediction rule for the severe acute respiratory syndrome Ann Intern Med 2005 142 225 15684216 10.7326/0003-4819-142-3-200502010-00019

[B41] Chow KY Lee CE Ling ML Heng DM Yap SG Outbreak of severe acute respiratory syndrome in a tertiary hospital in Singapore, linked to an index patient with atypical presentation: epidemiological study BMJ 2004 328 195 10.1136/bmj.37939.465729.44 14726369 PMC318482

[B42] Booth CM Matukas LM Tomlinson GA Rachlis AR Rose DB Dwosh HA Walmsley SL Mazzulli T Avendano M Derkach P Clinical features and short-term outcomes of 144 patients with SARS in the greater Toronto area JAMA 2003 289 2801 2809 10.1001/jama.289.21.JOC30885 12734147

[B43] Chan KH Poon LL Cheng VC Guan Y Hung IF Kong J Yam LY Seto WH Yuen KY Peiris JS Detection of SARS coronavirus in patients with suspected SARS Emerg Infect Dis 2004 10 294 299 15030700 10.3201/eid1002.030610 PMC3322905

[B44] Poon LL Chan KH Wong OK Yam WC Yuen KY Guan Y Lo YM Peiris JS Early diagnosis of SARS coronavirus infection by real time RT-PCR J Clin Virol 2003 28 233 238 10.1016/j.jcv.2003.08.004 14522060 PMC7129783

[B45] Liang W McLaws ML Liu M Mi J Chan DK Hindsight: a re-analysis of the severe acute respiratory syndrome outbreak in Beijing Public Health 2007 121 725 733 10.1016/j.puhe.2007.02.023 17555781 PMC7111616

[B46] Leung GM Lim WW Ho LM Lam TH Ghani AC Donnelly CA Anderson RM Seroprevalence of IgG antibodies to SARS-coronavirus in asymptomatic or subclinical population groups Epidemiol Infect 2003 134 211 221 10.1017/S0950268805004826 PMC2870380 16490123

