# Supplementary material for: A comparative epidemiologic analysis of SARS in Hong Kong, Beijing and Taiwan
Source: BMC Infect Dis. 2010 Mar 6;10:50. doi: 10.1186/1471-2334-10-50 (PMC2846944; doi:10.1186/1471-2334-10-50)
Supplement: Additional file 1 — Characteristics of SARS patients in Hong Kong, Beijing and Taiwan. The associated case-fatality ratios and adjusted odds ratios (95% confidence intervals) are also reported. CFR, case fatality ratio; AOR, adjusted odds ratio; CI, confidence interval. * Patients with unknown age, pre-existing comorbid conditions or admission date were excluded. † Adjusted for sex, age, health care worker status, preexisting comorbid conditions and nosocomial infection. ‡ Data on final outcome were not available for 12 patients in Taiwan and were excluded for analysis. § The estimates were not shown as there was not more than 2 deaths in these age groups out of a relatively large number of patients. || Based on the WHO World Standard Population distribution [16]. [file 1471-2334-10-50-S1.DOC]

**Additional file 1. Characteristics of SARS patients in Hong Kong, Beijing and Taiwan.**

|  |  | **Hong Kong (n=1755)** | | | | |  | **Beijing (n=917)** | | | | |  | **Taiwan (n=664)^‡^** | | | | |
| --- | --- | --- | --- | --- | --- | --- | --- | --- | --- | --- | --- | --- | --- | --- | --- | --- | --- | --- |
| Characteristic |  | No. of patients (%)^*^ | | CFR (%) | AOR**^†^** (95% CI) | |  | No. of patients (%) | | CFR (%) | AOR (95% CI) | |  | No. of patients (%) | | CFR (%) | AOR (95% CI) | |
| **Sex** |  |  |  |  |  |  |  |  |  |  |  |  |  |  |  |  |  |  |
| Female |  | 978 | (56) | 13.2 | 1 |  |  | 405 | (44) | 3.0 | 1 |  |  | 341 | (52) | 22.6 | 1 |  |
| Male |  | 777 | (44) | 22.3 | 1.40 | (1.01-1.94) |  | 512 | (56) | 3.5 | 0.85 | (0.38-1.87) |  | 311 | (48) | 33.1 | 1.17 | (0.79-1.74) |
| **Age group (years)** |  |  |  |  |  |  |  |  |  |  |  |  |  |  |  |  |  |  |
| 0-30 |  | 515 | (29) | 0.6 | 0.05 | (0.01-0.18) |  | 440 | (48) | 0.2 | n/a**^‡^** | |  | 170 | (26) | 13.5 | 0.74 | (0.39-1.42) |
| 31-40 |  | 379 | (22) | 7.1 | 0.68 | (0.38-1.20) |  | 193 | (21) | 1.0 | n/a**^‡^** | |  | 101 | (15) | 10.9 | 0.55 | (0.25-1.22) |
| 41-50 |  | 320 | (18) | 11.6 | 1 |  |  | 151 | (17) | 2.6 | 1 |  |  | 121 | (19) | 19.8 | 1 |  |
| 51-60 |  | 170 | (10) | 17.6 | 1.46 | (0.83-2.60) |  | 76 | (8.3) | 11.8 | 7.72 | (2.37-25.2) |  | 78 | (12) | 42.3 | 2.59 | (1.33-5.04) |
| 60+ |  | 371 | (21) | 55.3 | 5.63 | (3.52-9.01) |  | 57 | (6.2) | 24.6 | 8.52 | (2.36-30.8) |  | 181 | (28) | 48.6 | 2.94 | (1.66-5.21) |
| **Health Care Worker** |  |  |  |  |  |  |  |  |  |  |  |  |  |  |  |  |  |  |
| No |  | 1350 | (77) | 21.8 | 1 |  |  | 773 | (84) | 3.6 | 1 |  |  | 535 | (82) | 30.7 | 1 |  |
| Yes |  | 405 | (23) | 2.0 | 0.26 | (0.12-0.59) |  | 144 | (16) | 1.4 | 1.55 | (0.46-5.20) |  | 117 | (18) | 13.7 | 0.79 | (0.42-1.48) |
| **Preexisting comorbid conditions** |  | |  |  |  |  |  |  |  |  |  |  |  |  |  |  |  |  |
| No |  | 1395 | (80) | 10.0 | 1 |  |  | 755 | (96) | 1.5 | 1 |  |  | 455 | (70) | 22.2 | 1 |  |
| Yes |  | 358 | (20) | 45.5 | 1.59 | (1.09-2.31) |  | 35 | (4.4) | 14.3 | 4.06 | (1.62-10.19) |  | 197 | (30) | 40.1 | 1.64 | (1.09-2.46) |
| **Admitted before symptom onset** |  | |  |  |  |  |  |  |  |  |  |  |  |  |  |  |  |  |
| No |  | 1636 | (93) | 14.6 | 1 |  |  | 859 | (94) | 3.5 | 1 |  |  | 598 | (92) | 23.9 | 1 |  |
| Yes |  | 119 | (6.8) | 52.9 | 1.45 | (0.88-2.38) |  | 58 | (6.3) | 0.0 | n/a**^§^** | |  | 50 | (7.7) | 70.0 | 4.85 | (2.46-9.53) |
|  |  |  |  |  |  |  |  |  |  |  |  |  |  |  |  |  |  |  |
| **Deaths / Crude CFR (95% CI)** |  | 302 |  | 17.2 | (15.4, 19.0) | |  | 30 |  | 3.3 | (2.1, 4.4) | |  | 180 |  | 27.6 | (24.2, 31.0) | |
| **Age-sex standardized CFR^║^** |  |  |  | 10.6 | (9.2, 12.2) | |  |  |  | 4.5 | (2.8, 6.7) | |  |  |  | 18.8 | (15.2, 22.9) | |

The associated case-fatality ratios and adjusted odds ratios (95% confidence intervals) are also reported.

**CFR, case fatality ratio; AOR, adjusted odds ratio; CI, confidence interval.**

**^*^ Patients with unknown age, pre-existing comorbid conditions or admission date were excluded.**

**^†^ Adjusted for sex, age, health care worker status, preexisting comorbid conditions and nosocomial infection.**

**^‡^ Data on final outcome were not available for 12 patients in Taiwan and were excluded for analysis.**

**^§^ The estimates were not shown as there was not more than 2 deaths in these age groups out of a relatively large number of patients.**

**^║║^ Based on the WHO World Standard Population distribution [16].**
