# Supplementary material for: A comparative epidemiologic analysis of SARS in Hong Kong, Beijing and Taiwan
Source: BMC Infect Dis. 2010 Mar 6;10:50. doi: 10.1186/1471-2334-10-50 (PMC2846944; doi:10.1186/1471-2334-10-50)
Supplement: Additional file 2 — Characteristics of SARS patients in Hong Kong, Beijing and Taiwan (pooled data). The associated case-fatality ratios and adjusted odds ratios (95% confidence intervals) are also reported. CFR, case fatality ratio; AOR, adjusted odds ratio; CI, confidence interval. * Data on final outcome were not available for 12 patients in Taiwan and were excluded for analysis. Patients with unknown age, pre-existing comorbid conditions or admission date were excluded from multivariable logistic regression models. † Adjusted for sex, age, health care worker status, preexisting comorbid conditions, nosocomial infection and region. ‡ Based on the WHO World Standard Population distribution [16]. [file 1471-2334-10-50-S2.DOC]

**Additional file 2. Characteristics of SARS patients in Hong Kong, Beijing and Taiwan (pooled data).**

|  |  | **All three regions (n=3336)^*^** | | | | |
| --- | --- | --- | --- | --- | --- | --- |
| Characteristic |  | No. of patients (%)**^*^** | | CFR (%) | AOR**^†^** (95% CI) | |
|  |  |  |  |  |  |  |
| **Sex** |  |  |  |  |  |  |
| Female |  | 1724 | (52) | 12.7 | 1 |  |
| Male |  | 1600 | (48) | 18.4 | 1.24 | (1.00-1.55) |
| **Age group (years)** |  |  |  |  |  |  |
| 0-30 |  | 1125 | (34) | 2.4 | 0.27 | (0.18-0.43) |
| 31-40 |  | 673 | (20) | 5.9 | 0.61 | (0.41-0.92) |
| 41-50 |  | 592 | (18) | 11.0 | 1 |  |
| 51-60 |  | 324 | (10) | 22.2 | 2.10 | (1.45-3.04) |
| 60+ |  | 609 | (18) | 50.4 | 4.57 | (3.32-7.30) |
| **Health Care Worker** |  |  |  |  |  |  |
| No |  | 2658 | (80) | 18.3 | 1 |  |
| Yes |  | 666 | (20) | 3.9 | 0.49 | (0.32-0.74) |
| **Preexisting comorbid conditions** | | |  |  |  |  |
| No |  | 2605 | (81) | 9.7 | 1 |  |
| Yes |  | 590 | (19) | 41.8 | 1.74 | (1.36-2.21) |
| **Admitted before symptom onset** | | |  |  |  |  |
| No |  | 3093 | (93) | 13.3 | 1 |  |
| Yes |  | 227 | (6.8) | 43.2 | 2.07 | (1.47-2.91) |
| **Region** |  |  |  |  |  |  |
| Hong Kong |  | 1755 | (53) | 17.2 | 1 |  |
| Beijing |  | 917 | (28) | 3.3 | 0.18 | (0.11-0.29) |
| Taiwan |  | 642 | (19) | 27.5 | 1.64 | (1.29-2.07) |
|  |  |  |  |  |  |  |
| **Deaths / Crude CFR** (95% CI) |  | 512 |  | 15.4 | (14.2, 16.7) | |
| **Age-sex standardized CFR^‡^** (95% CI) | | |  | 10.7 | (9.6, 11.9) | |

The associated case-fatality ratios and adjusted odds ratios (95% confidence intervals) are also reported.

**CFR, case fatality ratio; AOR, adjusted odds ratio; CI, confidence interval.**

**^*^ Data on final outcome were not available for 12 patients in Taiwan and were excluded for analysis. Patients with unknown age, pre-existing comorbid conditions or admission date were excluded from multivariable logistic regression models.**

**^†^ Adjusted for sex, age, health care worker status, preexisting comorbid conditions, nosocomial infection and region.**

**^‡^ Based on the WHO World Standard Population distribution [16]**
