# Supplementary material for: A comparative epidemiologic analysis of SARS in Hong Kong, Beijing and Taiwan
Source: BMC Infect Dis. 2010 Mar 6;10:50. doi: 10.1186/1471-2334-10-50 (PMC2846944; doi:10.1186/1471-2334-10-50)
Supplement: Additional file 3 — Case fatality ratio by different onset-to-admission periods, Hong Kong, Beijing and Taiwan. CFR, case fatality ratio; CI, confidence interval. * Excluding 16 patients with unknown admission dates or discharge outcome. [file 1471-2334-10-50-S3.DOC]

**Additional file 3. Case fatality ratio by different onset-to-admission periods, Hong Kong, Beijing and Taiwan**

|  |  | **Hong Kong (n = 1755)** | | | |  | **Beijing (n = 917)** | | | |  | **Taiwan (n = 648*)** | | | |
| --- | --- | --- | --- | --- | --- | --- | --- | --- | --- | --- | --- | --- | --- | --- | --- |
| Delay from onset to admission |  | Patients, n (%) | | CFR, % (95% CI) | |  | Patients, n (%) | | CFR, % (95% CI) | |  | Patients, n (%) | | CFR, % (95% CI) | |
| Admitted before symptom onset date |  | 119 | (6.8) | 52.9 | (44.0, 61.7) |  | 58 | (6.3) | 0 | (0, 6.2) |  | 50 | (7.7) | 70.0 | (14.7, 94.7) |
| 0-1 days |  | 392 | (22.3) | 19.1 | (15.5, 23.3) |  | 477 | (52.0) | 2.1 | (1.1, 3.8) |  | 264 | (40.7) | 27.3 | (22.0, 33.1) |
| 2-3 days |  | 545 | (31.1) | 14.1 | (11.5, 17.3) |  | 159 | (17.3) | 4.4 | (2.1, 8.8) |  | 144 | (22.2) | 26.4 | (19.4, 34.4) |
| 4-5 days |  | 357 | (20.3) | 11.2 | (8.3, 14.9) |  | 81 | (8.9) | 7.4 | (3.4, 15.2) |  | 97 | (15.0) | 14.4 | (8.1, 23.0) |
| 6-7 days |  | 194 | (11.1) | 14.4 | (10.2, 20.1) |  | 60 | (6.6) | 5.0 | (1.7, 13.7) |  | 54 | (8.3) | 16.7 | (7.9, 29.3) |
| ≥8 days |  | 148 | (8.4) | 12.8 | (8.4, 19.2) |  | 82 | (8.9) | 4.9 | (1.9, 11.9) |  | 39 | (6.1) | 25.6 | (13.0, 42.1) |

**CFR, case fatality ratio; CI, confidence interval.**

**^*^ Excluding 16 patients with unknown admission dates or discharge outcome.**
