# Supplementary material for: A comparative epidemiologic analysis of SARS in Hong Kong, Beijing and Taiwan
Source: BMC Infect Dis. 2010 Mar 6;10:50. doi: 10.1186/1471-2334-10-50 (PMC2846944; doi:10.1186/1471-2334-10-50)
Supplement: Additional file 4 — Case fatality ratio by different onset-to-admission periods in Beijing, XTS Hospital, Hospital 302 and Hospital 309. CFR, case fatality ratio; CI, confidence interval. [file 1471-2334-10-50-S4.DOC]

**Additional file 4:** Case fatality ratio by different onset-to-admission periods in Beijing, XTS Hospital, Hospital 302 and Hospital 309

|  |  | **XTS Hospital (n=680)** | | | |  | **Hospital 302 (n=111)** | | | |  | **Hospital 309 (n=126)** | | | |
| --- | --- | --- | --- | --- | --- | --- | --- | --- | --- | --- | --- | --- | --- | --- | --- |
| Delay from onset to admission |  | Patients, n (%) | | CFR, % (95% CI) | |  | Patients, n (%) | | CFR, % (95% CI) | |  | Patients, n (%) | | CFR, % (95% CI) | |
| Admitted before symptom onset date |  | 57 | (8.4) | 0 | (0, 6.3) |  | 1 | (0.9) | 0 | (0, 94.9) |  | 0 | (0) | n/a | |
| 0-1 days |  | 412 | (60.6) | 1.2 | (0.5, 2.8) |  | 49 | (44.1) | 6.1 | (2.1, 16.5) |  | 16 | (12.7) | 12.5 | (3.5, 36.0) |
| 2-3 days |  | 114 | (16.8) | 1.8 | (0.5, 6.2) |  | 26 | (23.4) | 7.7 | (2.1, 24.1) |  | 19 | (15.1) | 15.8 | (5.5, 37.6) |
| 4-5 days |  | 40 | (5.9) | 2.5 | (0.1, 12.9) |  | 12 | (10.8) | 16.7 | (4.7, 44.8) |  | 29 | (23.0) | 10.3 | (3.6, 26.4) |
| 6-7 days |  | 25 | (3.7) | 0 | (0, 13.3) |  | 9 | (8.1) | 11.1 | (0.6, 43.5) |  | 26 | (20.6) | 7.7 | (2.1, 24.1) |
| ≥8 days |  | 32 | (4.7) | 0 | (0, 10.7) |  | 14 | (12.6) | 0 | (0, 21.5) |  | 36 | (28.6) | 11.1 | (4.4, 25.3) |

**CFR, case fatality ratio; CI, confidence interval.**
