# Supplementary material for: A comparative epidemiologic analysis of SARS in Hong Kong, Beijing and Taiwan
Source: BMC Infect Dis. 2010 Mar 6;10:50. doi: 10.1186/1471-2334-10-50 (PMC2846944; doi:10.1186/1471-2334-10-50)
Supplement: Additional file 6 — Factors affecting the onset-to-death and onset-to-discharge period of SARS patients in Hong Kong, Beijing (restricted to Hospitals 302 and 309 only and Taiwan. CI, confidence interval. * The acceleration factor is computed as exp(β). It indicates the relative increase (>1) or decrease (<1) in the median time from onset of symptoms to death or discharge. † also adjusted for interaction between location with admission before symptom onset. ‡ also adjusted for interaction between location with health care worker and pre-existing comorbid conditions. [file 1471-2334-10-50-S6.DOC]

**Additional file 6: Factors affecting the onset-to-death and onset-to-discharge period of SARS patients in Hong Kong, Beijing (restricted to Hospitals 302 and 309 only) and Taiwan**

|  |  | **Acceleration Factor (exp(β))^*^** | | | | |
| --- | --- | --- | --- | --- | --- | --- |
|  |  | **Onset-to-death^†^** | |  | **Onset-to-discharge^‡^** | |
| Characteristic |  | Estimate | (95% CI) |  | Estimate | (95% CI) |
|  |  |  |  |  |  |  |
| **Sex** |  |  |  |  |  |  |
| Female |  | 1 |  |  | 1 |  |
| Male |  | 1.04 | (0.91-1.18) |  | 1.00 | (0.95-1.05) |
|  |  |  |  |  |  |  |
| **Age group (years)** |  |  |  |  |  |  |
| 0-30 |  | 0.61 | (0.43-0.85) |  | 0.87 | (0.81-0.94) |
| 31-40 |  | 0.85 | (0.63-1.15) |  | 0.93 | (0.86-1.00) |
| 41-50 |  | 1 |  |  | 1 |  |
| 51-60 |  | 0.87 | (0.67-1.13) |  | 1.03 | (0.93-1.14) |
| 60+ |  | 0.77 | (0.62-0.95) |  | 1.00 | (0.91-1.10) |
|  |  |  |  |  |  |  |
| **Health Care Worker** |  |  |  |  |  |  |
| No |  | 1 |  |  | 1 |  |
| Yes |  | 1.44 | (1.06-1.96) |  | 0.98 | (0.92-1.05) |
|  |  |  |  |  |  |  |
| **Preexisting comorbid conditions** | | |  |  |  |  |
| No |  | 1 |  |  | 1 |  |
| Yes |  | 0.85 | (0.74-0.98) |  | 0.96 | (0.87-1.05) |
|  |  |  |  |  |  |  |
| **Admitted before symptom onset** | | |  |  |  |  |
| No |  | 1 |  |  | 1 |  |
| Yes |  | 0.62 | (0.50-0.77) |  | 1.08 | (0.94-1.24) |
|  |  |  |  |  |  |  |
| **Region** |  |  |  |  |  |  |
| Hong Kong |  | 1 |  |  | 1 |  |
| Beijing |  | 0.87 | (0.51-1.47) |  | 1.72 | (1.47-2.00) |
| Taiwan |  | 0.43 | (0.36-0.50) |  | 0.75 | (0.70-0.81) |

**CI, confidence interval.**

**^*^ The acceleration factor is computed as exp(β). It indicates the relative increase (>1) or decrease (<1) in the median time from onset of symptoms to death or discharge.**

**^†^ adjusted for sex, age, health care worker status, preexisting comorbid conditions, nosocomial infection and region, also adjusted for significant interaction (based on Wald test) between location with admission before symptom onset.**

**^‡^ adjusted for sex, age, health care worker status, preexisting comorbid conditions, nosocomial infection and region, also adjusted for significant interaction (based on Wald test) between location with health care worker and pre-existing comorbid conditions.**
